# Application of a chain management protocol in patients with chronic refractory wounds under the medical consortium model: a health-services intervention study

**DOI:** 10.3389/fpubh.2026.1837193

**Published:** 2026-09-11

**Authors:** Yan Hu, Qin He, Fazhen Yu, Ruyu Huang, Danting Zhu, Hui Tu, Xueqin Li, Bei Zhao, Qingxia Hu

**Affiliations:** 1 Wound and Ostomy Clinic, The Second Affiliated Hospital of Nanchang University, Nanchang, China; 2 Nursing Department, The Second Affiliated Hospital of Nanchang University, Nanchang, China

**Keywords:** chain management, chronic refractory wounds, continuity of care, health service delivery, medical consortium, multidisciplinary collaboration

## Abstract

**Background/objective:**

Chronic refractory wounds represent a major public health challenge in China, characterized by fragmented and unstandardized services, insufficient continuity of care, and weak inter-institutional collaboration. This study aimed to evaluate the effectiveness of a medical consortium–based chain management protocol for improving health service quality, care continuity, and clinical outcomes in chronic wound management.

**Methods:**

This was a health services intervention study conducted at a tertiary hospital in Nanchang. From June 2024 to May 2025, 68 patients with chronic wounds were enrolled using convenience sampling and randomly allocated to intervention (*n* = 34) or control (*n* = 34) groups. The control group received conventional care, while the intervention group received a structured chain management protocol including multidisciplinary team (MDT) establishment, standardized care pathways, homogeneous training, and full-cycle coordinated care from hospital treatment to post-discharge linkage with primary care facilities. Outcome measures included Bates-Jensen Wound Assessment Tool scores, wound healing time, referral and consultation efficiency, and patient satisfaction.

**Results:**

Repeated-measures ANOVA showed a significant time–group interaction for Bates-Jensen scores (*F* = 4.075, *p* = 0.010). Bates-Jensen scores are reported as median (P25, P75); non-parametric sensitivity analyses confirmed no baseline difference between groups, with the intervention group showing progressively lower scores, most markedly at Week 4 (*p* < 0.001). Median healing time was 30.5 days in the intervention group versus 52.5 days in the control group (*p* = 0.001). Patient satisfaction was 97.1% in the intervention group compared with 76.5% in the control group (*p* = 0.027). The intervention also improved inter-institutional referral and consultation efficiency.

**Conclusion:**

A medical consortium–based chain management protocol effectively optimizes health service organization, strengthens interdisciplinary and inter-institutional collaboration, and improves the continuity, standardization, and efficiency of chronic wound care. This model shortens healing time, enhances patient satisfaction, and provides an implementable and scalable strategy for chronic wound management in the health service system. It offers important implications for public health practice and resource allocation in wound care management.

## 1 Introduction

Chronic wounds refer to wounds that fail to proceed through the healing process normally and show no significant progress after more than 1 month (1, 2). Common types include venous ulcers, diabetic foot ulcers, pressure injuries, and arterial ulcers (3, 4). Globally, with the aging of the population and the rising prevalence of chronic diseases such as diabetes and cardiovascular disorders, chronic wounds have evolved into an increasingly severe public health challenge. The pooled prevalence rate reaches 2.21 cases per 1,000 population, among which chronic leg ulcers account for the largest proportion, with a prevalence of 1.51 cases per 1,000 population (1). In the United States, approximately 2% of the population is affected, corresponding to a total of over 6 million patients (5). These wounds not only cause long-term pain and functional limitations in patients but also significantly reduce their quality of life, particularly in terms of physical function and pain levels (6). Notably, the 3-year recurrence rate of diabetic foot ulcers is as high as 64.4% (7). Meanwhile, chronic wounds impose substantial medical burdens: in developed countries, their treatment costs account for approximately 1–3% of total healthcare expenditures. Annual related costs in the United States have exceeded 37 billion United States dollars (6), and the median treatment cost for a single case of diabetes-related chronic wound in New Zealand is about 22,400 New Zealand dollars (8). Frequent hospitalizations and long-term care further consume enormous medical and health resources, placing significant strain on the global healthcare economic system (4).

In China, the burden of chronic wounds has grown in tandem with rapid population aging and a surging diabetes epidemic. By the end of 2023, 296.97 million Chinese residents (21.1% of the total population) were aged 60 years or older (9), and the International Diabetes Federation estimated that 140.9 million Chinese adults aged 20–79 years had diabetes in 2021—the highest number of any country worldwide (10). Serial multi-center epidemiological surveys have documented a corresponding etiological shift: whereas trauma and infection predominated in 1998 (diabetes accounting for only 4.91% of cases) (11), diabetes had become the single leading cause of chronic cutaneous wounds by 2007-2008 (31.3% of male and 35.3% of female patients) (12), a pattern further confirmed in a 2018 update across 17 tertiary hospitals (13). Overall, it has been estimated that chronic wounds affect approximately 50 million people in China annually (14), and inpatient data from Sichuan Province showed that the median cost of care for patients with complex wounds (¥6,500) was nearly double the all-cause median (¥3,337) (15). These trends highlight an urgent need for integrated, systematic wound-management models tailored to China’s healthcare structure.

Meeting this need, however, is challenging. The traditional model exhibits obvious fragmented characteristics, such as poor intra-hospital interdisciplinary collaboration, interrupted post-discharge transitional care, and the lack of a sound management system and mutual recognition mechanism for medical information within medical consortia. Primary healthcare institutions suffer from a shortage of nursing resources and insufficient specialized competencies of nurses. Meanwhile, patients’ lack of trust and rigid perceptions hinder the implementation of hierarchical diagnosis and treatment, resulting in poor continuity of patient care and low treatment efficiency (16). To address this dilemma, China has promoted hierarchical diagnosis and treatment with the construction of medical consortia as a crucial carrier. By vertically integrating medical resources from tertiary hospitals, community health service centers, and other institutions, a collaborative division of labor featuring “treating minor illnesses in communities, severe illnesses in hospitals, and conducting rehabilitation back in communities” has been established (17), providing key policy support and organizational foundation for chronic wound management. Through hospital-community linkage, two-way referral, and technology outreach, medical consortia have standardized the hierarchical management process of chronic wounds and improved the service capacity of primary institutions.

Against this backdrop, exploring effective wound management practices based on medical consortia is of great importance. Multidisciplinary Team (MDT) collaboration is widely recognized as a core strategy for managing complex chronic wounds. By integrating the expertise of surgery, endocrinology, nutrition, rehabilitation, and other disciplines to formulate comprehensive treatment plans, MDT can not only significantly shorten the healing time of chronic wounds such as diabetic foot ulcers and reduce the incidence of in-hospital complications (18) but also effectively lower the risk of hospital-acquired pressure injuries, shorten the length of hospital stay, and optimize treatment outcomes (19). However, how to apply the MDT concept to the cross-institutional scenarios of medical consortia and form a seamlessly connected, standardized operational pathway remains a gap in practice. Chain management, as a management method emphasizing closed-loop processes, interlocking links, and full-process quality control, provides a valuable theoretical perspective for designing such pathways (20, 21). By establishing clear responsibility chains and referral criteria, it is expected to ensure that patients receive consistent and appropriate services at every stage of diagnosis and treatment.

At present, although a few studies have explored the application of wound clinic-led nursing pathways or in-hospital MDT models (5, 22), there is a lack of rigorous evidence on the effectiveness of a standardized management scheme that deeply integrates chain management with the medical consortium structure, with wound, ostomy, and continence nurses as core coordinators, covering both in-hospital and out-of-hospital settings and running through the entire care continuum. In particular, whether this model can significantly accelerate wound healing, optimize medical resource circulation, and improve patient experience still needs to be verified through controlled studies. The objective of this study was to evaluate the effectiveness of a medical consortium–based chain management protocol, compared with conventional wound care, in improving wound status, shortening healing time, and enhancing care continuity and patient satisfaction. We hypothesized that patients managed under the chain management protocol would demonstrate greater improvement in wound status (Bates-Jensen Wound Assessment scores), shorter wound healing time, and higher patient satisfaction than those receiving conventional care.

## 2 Methods

### 2.1 Study design and setting

This was a single-center, prospective health services intervention study with a concurrent control group, conducted at the Wound and Ostomy Clinic of the Second Affiliated Hospital of Nanchang University, a tertiary Grade-A teaching hospital with two campuses in Nanchang, Jiangxi Province, China, with 3,516 beds and an annual outpatient volume of approximately 3.08 million visits. The hospital admits an average of approximately 15,000 patients with chronic wounds annually. The clinic, which serves as the hub of a regional medical consortium of 15 units for chronic wound care covering Nanchang and surrounding areas, is staffed by 6 full-time certified wound and ostomy specialist nurses and 8 rotating nurses. A prospective controlled design was chosen because it provides a rigorous concurrent comparison of the chain management protocol relative to conventional care while reflecting the real-world implementation of a care-delivery model within a medical consortium. The study was approved by the Biomedical Research Ethics Committee of the Second Affiliated Hospital of Nanchang University (Approval No.: I-MedResEthicsRev [2024] No. (50)).

Patients with chronic wounds treated at the hospital were recruited by convenience sampling from June 2024 to May 2025, and all eligible patients who provided written informed consent were randomly allocated at the individual level to the intervention group or the control group with equal allocation probability (1:1) using a coin-tossing method (see Section 2.3). To minimize contamination between groups, patients allocated to the control group and those allocated to the intervention group were attended in two separate consulting rooms (Consulting Room 1 and Consulting Room 2, respectively) of the Wound and Ostomy Clinic.

#### 2.1.1 Sample size

No *a priori* sample size calculation was performed. Patients were enrolled and randomly allocated at a 1:1 ratio over the 12-month study period, and recruitment closed at the end of this period, yielding 34 patients per group (total *N* = 68), reflecting a feasibility-based approach. A *post hoc* sensitivity analysis was performed using G*Power 3.1 to evaluate the achieved statistical power. For the primary outcome (Bates-Jensen score at Week 4), the observed between-group difference corresponded to a large effect size (Cohen’s *d* = 0.93); with 34 patients per group and a two-sided *α* of 0.05, the achieved power was 0.97. The study was thus adequately powered to detect the primary between-group effect, and was capable of detecting standardized effect sizes of *d* ≥ 0.69 with 80% power.

### 2.2 Study participants

#### 2.2.1 Inclusion criteria

Meets the diagnostic criteria for chronic wounds (healing process is impaired with no significant progress for more than 4 weeks); Aged 18–75 years; Understands the study content and voluntarily signs the informed consent form; Able to cooperate with full-course follow-up and related assessments.

#### 2.2.2 Exclusion criteria

Complicated with malignant tumors, tuberculosis, syphilis, Behçet’s disease, Crohn’s disease, severe autoimmune disorders, or other similar conditions; Complicated with acute failure of heart, liver, kidney, or other vital organs; Use of corticosteroids, immuno-suppressants, cytotoxic drugs, or other similar treatments within 3 months prior to enrollment; Presence of cognitive impairment or mental illness that prevents cooperation with interventions and data collection; Pregnant or lactating women; Uncontrolled severe infection at the wound site.

#### 2.2.3 Target wound selection

Each patient contributed a single target wound to the assessment and analysis. For patients presenting with multiple chronic wounds, the largest wound was designated as the target wound, and all wound-related outcome measures, including Bates-Jensen Wound Assessment Tool scores and wound healing time, were recorded for this target wound. The largest wound was selected because it typically represents the greatest clinical burden and healing challenge, and restricting the analysis to one wound per patient ensured the independence of observations and avoided potential clustering effects arising from multiple wounds within the same patient. This selection criterion was applied uniformly to patients in both groups at enrollment.

### 2.3 Randomization and blinding

Patients were randomly allocated to the control or intervention group using a coin-toss method at a 1:1 ratio; by chance, the two groups ended with an identical number of patients (34 each). Allocation was performed by an independent investigator who was not involved in patient care, follow-up, or outcome assessment, in order to minimize allocation bias. After allocation, each patient was directed to the consulting room corresponding to their assigned group; this physical separation of the two groups was implemented to prevent contamination, as patients in the intervention group received the chain management protocol while control patients received conventional care in a different room.

Due to the nature of the service intervention, blinding of patients and healthcare providers was not feasible, and the study was therefore conducted open-label. To limit potential assessment bias, wound status was evaluated using the Bates-Jensen Wound Assessment Tool, a standardized instrument with explicitly defined items and scoring criteria, and follow-up assessments were performed at pre-specified time points.

### 2.4 Intervention measures

#### 2.4.1 Control group

Conventional wound care was provided. After outpatient consultation, routine wound assessment, debridement, and dressing selection were performed according to the patient’s specific condition and the local wound status, and dressing changes were carried out at the clinic as clinically indicated. Routine guidance was given on diet, exercise, and precautions related to dressing changes. Conventional care did not include standardized MDT consultation, a medical-consortium-based two-way referral system, structured post-discharge follow-up, or homogeneous training and support from the tertiary hospital. For wounds that could not be managed in the outpatient clinic, patients were advised to self-refer for hospitalization; after discharge, patients self-managed their wounds without systematic linkage to primary-care institutions.

#### 2.4.2 Intervention group

A medical consortium-based chain management protocol was implemented, including the following components:

##### 2.4.2.1 Establishment of a medical consortium chain management team

The team consisted of an in-hospital Multidisciplinary Team (MDT) and an out-of-hospital medical consortium team.

- *In-hospital MDT*: Composed of departments including the Wound and Ostomy Clinic, Endocrinology Department, Orthopedics Department, Wound Repair Department, Pain Management Department, Nutrition Department, and Clinical Laboratory, with 17core members and 12 medical expert consultants. Core members included 1 chief physician, 2 attending physicians, 6 full-time wound and ostomy nurses, and 8 rotating nurses, responsible for initial consultation, specialist consultation, and follow-up of chronic wounds. Through inter-departmental collaboration within the hospital, rapid and effective diagnosis and treatment services were provided to ensure the continuity of wound management. The medical expert consultant group consisted of attending physicians from each department who assisted the MDT in diagnosis and treatment and provided further management for patients referred from outpatient clinics or lower-level hospitals.
- *Out-of-hospital medical consortium team*: Composed of core members and 15 closely affiliated medical consortium hospitals, covering Nanchang urban area and surrounding regions including Jiujiang, Jingdezhen, Yingtan, Anyi County, and Wuning County. To date, the Wound and Ostomy Clinic has signed agreements on continuous care for chronic wounds with 15 wound and ostomy specialist nurses from these lower-level medical consortium hospitals, who serve as liaison officers to coordinate the diagnosis and treatment of complex and refractory wound patients with the specialist nurses of the hospital, enabling two-way referral and continuous care.

##### 2.4.2.2 Development of a medical consortium chain management implementation process core

**Figure 1:**
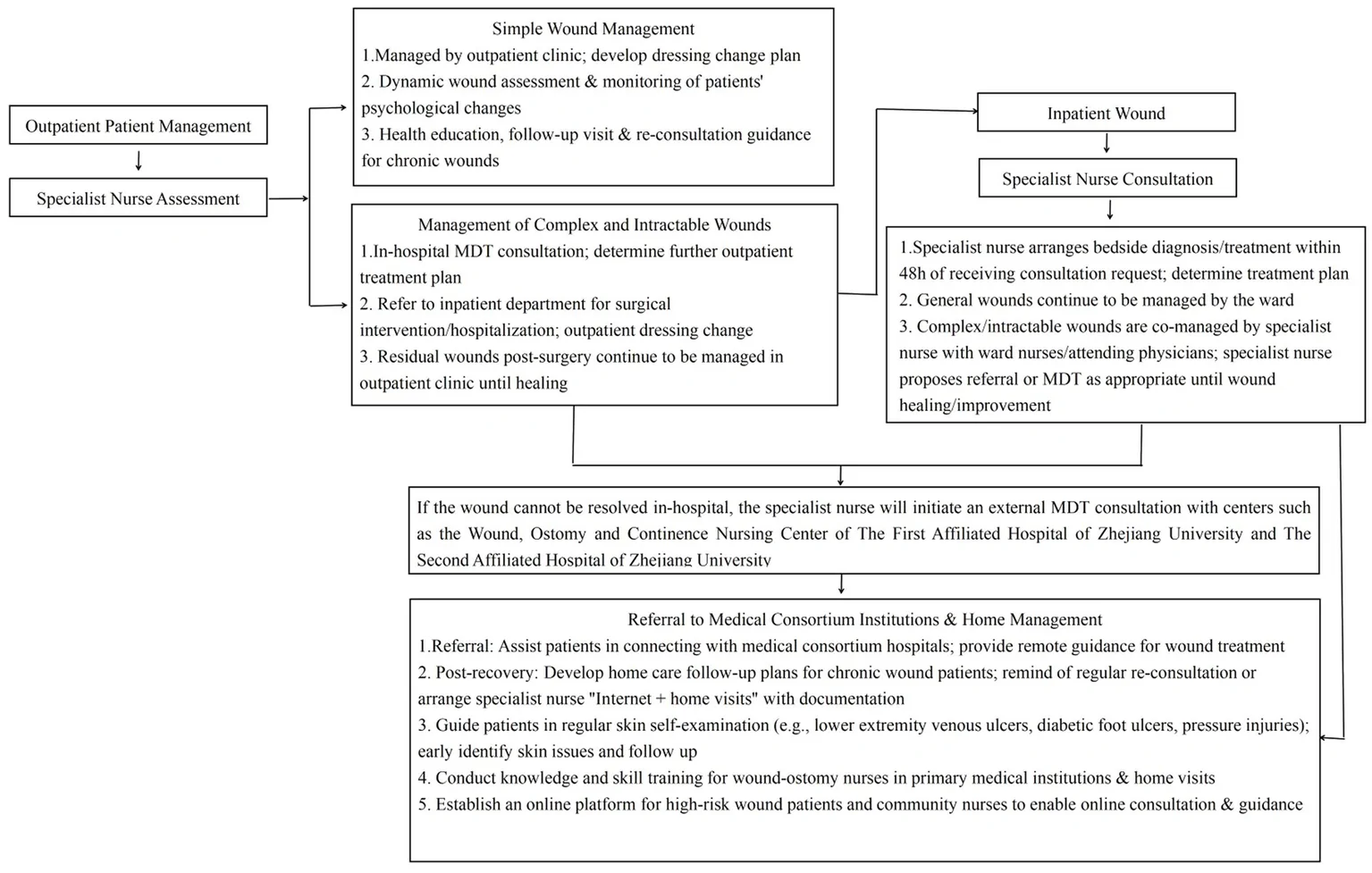
In-hospital chain flow chart.

**Figure 2:**
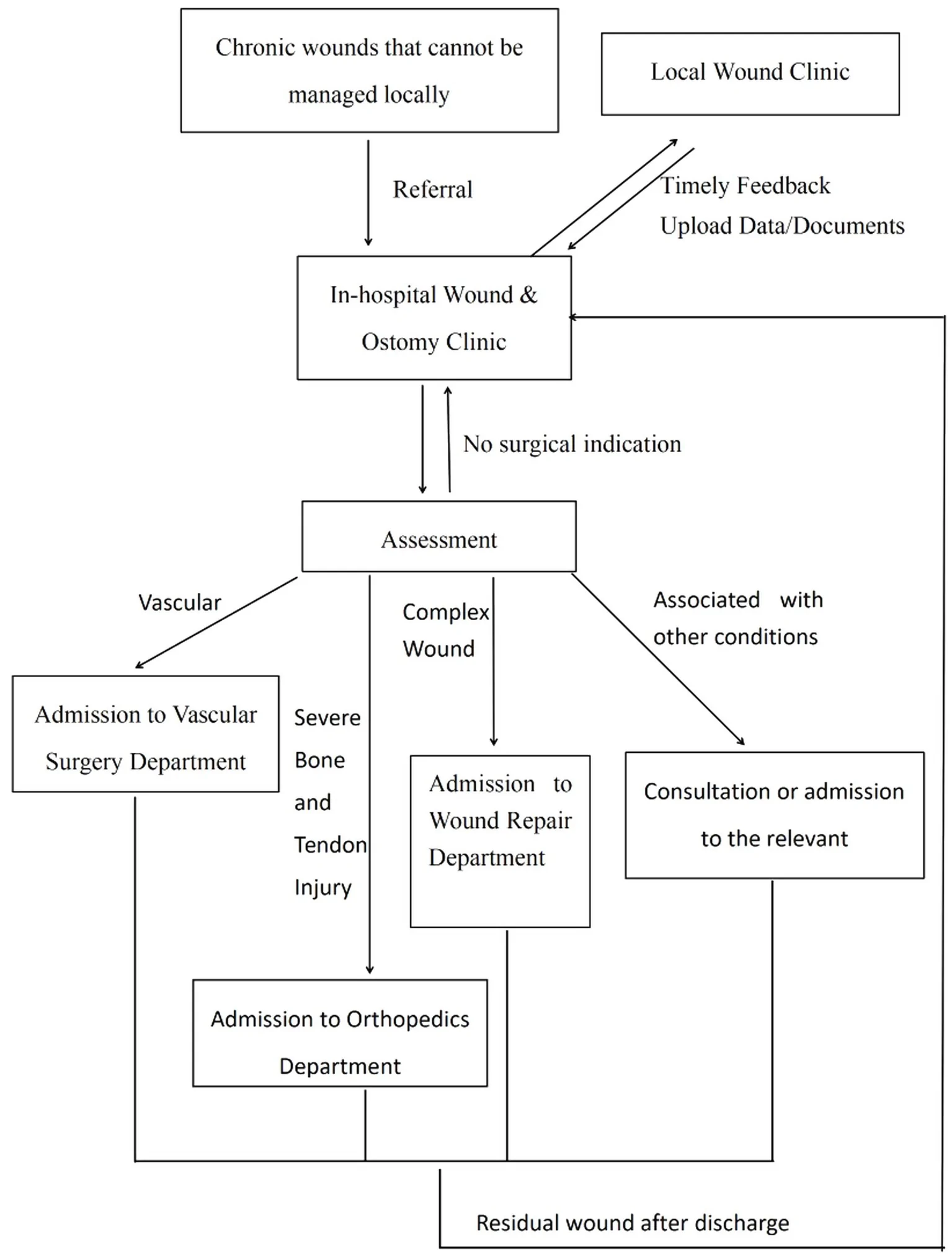
Out-of-hospital chain flow chart.

members and the MDT expert consultant group systematically identified in-hospital and out-of-hospital challenges in the diagnosis and treatment of chronic refractory wounds, including: ① Insufficient experience of inpatient physicians and ward nurses in managing complex wounds, with a need for improved dressing change techniques; ② For some discharged patients with wounds returning to their local areas, treatment progress could not be tracked, resulting in interrupted continuous wound care and low patient satisfaction; ③ Healthcare providers in primary hospitals lacked access to updated knowledge on wound management and had inadequate ability to assess and treat chronic refractory wounds; ④ Poor communication between medical staff in primary and higher-level hospitals led to delayed treatment and referral of local chronic wound patients, missing the optimal treatment window. Based on these challenges, combined with the latest literature and clinical practice, the core members and MDT expert consultant group jointly developed the medical consortium chain management implementation process, as detailed in Figures 1 and 2.

##### 2.4.2.3 Training of medical consortium chain management team members

To ensure the smooth implementation of the chain management protocol, homogeneous training was provided to MDT members and liaison officers from medical consortium hospitals, including:

- *Theoretical and skill training*: Conduct offline intensive theoretical training through national and provincial continuing medical education programs, and regularly/irregularly push learning materials via online platforms to comprehensively improve the professional theories and skills of nurses in our hospital and medical alliance hospitals.
- *Online guidance*: Members of medical consortium units can obtain team guidance through the platform when encountering complex wound cases.
- *On-site guidance*: Team experts conducted on-site theoretical lectures and practical operation demonstrations in primary hospitals.

##### 2.4.2.4 Key implementation points of medical consortium chain management

After the Wound and Ostomy Clinic directly admitted or received referrals of chronic refractory wound patients from lower-level medical consortium units, specialist nurses first assessed the patient’s wound condition and general health status, with the following key implementation points:① Formulating dressing plans for manageable wounds and coordinating referrals for comorbidities; ② Initiating in-hospital MDT consultation and green-channel hospitalization for patients requiring surgery or systemic treatment; ③ Responding to ward consultations within 24 h and co-managing complex wounds; ④ Requesting out-of-hospital MDT consultation for unresolved cases; ⑤ Facilitating post-discharge linkage with medical consortium hospitals and providing remote guidance; ⑥ Developing home care follow-up plans and providing “Internet + home-based nursing”; ⑦ Conducting regular training and home visits for primary nurses and establishing an online communication platform.

### 2.5 Outcome measures

#### 2.5.1 Primary outcome measures

##### 2.5.1.1 Wound treatment efficacy

Assessed using the Chinese version of the Bates-Jensen Wound Assessment Tool (BWAT), which comprises 8 items (wound size, undermining extent, necrotic tissue type, necrotic tissue amount, exudate type, exudate amount, granulation tissue status, and epithelialization status). size and undermining scored from 0 (healed) to 5, other items from 1 to 5, with a total score ranging from 6 to 40; higher scores indicate more severe wound conditions, while lower scores reflect better wound healing outcomes. The Chinese version of the instrument has demonstrated good reliability (Cronbach’s α = 0.86). Assessments were performed at baseline (Initial visit) and at 1, 2, and 4 weeks after the intervention.

##### 2.5.1.2 Wound healing time

The duration from the first dressing change and debridement to complete wound healing (epithelial tissue fully covers the wound, with no exudate or signs of infection).

#### 2.5.2 Secondary outcome measures

##### 2.5.2.1 Referral and consultation status

The number of home visits, on-site consultations, remote consultations by wound specialist nurses, and the number of patients referred within the medical consortium (including both referrals from lower-level hospitals to our center and referrals from our center back to lower-level hospitals) were recorded throughout the intervention period.

##### 2.5.2.2 Patient satisfaction

Evaluated using a questionnaire designed by the research team specifically for this study, developed based on the key service dimensions of the chain management protocol. The questionnaire covers 5 dimensions (overall satisfaction, consultation process, proactive service awareness, technical operation level, and continuous care), each rated on a 5-point scale (“very dissatisfied,” “dissatisfied,” “relatively satisfied,” “satisfied,” and “very satisfied”), with ratings of “relatively satisfied or above” categorized as satisfactory. Assessments were conducted at the end of the intervention.

### 2.6 Data collection methods

Data were collected by three of the clinic’s trained wound and ostomy specialist nurses using a general information questionnaire, the Bates-Jensen Wound Assessment Tool (described in Section 2.5.1), consultation/referral records, and a patient satisfaction questionnaire. Baseline data were collected at enrollment; wound assessments were performed at 1, 2, and 4 weeks; satisfaction data were collected at the end of the intervention.

### 2.7 Statistical methods

SPSS 23.0 software was used for statistical analysis. The normality of continuous variables was assessed using the Shapiro–Wilk test. Normally distributed measurement data were described as mean ± standard deviation (*x* ± *s*), and comparisons between groups were performed using independent samples *t*-test. Non-normally distributed measurement data were expressed as median (P25, P75), and comparisons between groups were conducted using the Mann–Whitney *U* test. Categorical data were described as frequency and percentage (%), and comparisons between groups were performed using the *χ*^2^ test or Fisher’s exact test. Two-way repeated-measures analysis of variance (ANOVA) was used to compare Bates-Jensen Wound Assessment Tool scores between the two groups at different time points; given the equal group sizes (*n* = 34 per group), the ANOVA was retained as the primary analysis owing to its robustness to moderate departures from normality. As the Week-4 Bates-Jensen scores departed from normality in both groups (Shapiro–Wilk *p* < 0.05), these scores are presented as median (P25, P75), and Mann–Whitney *U* tests at each time point were performed as non-parametric sensitivity analyses (Bonferroni-adjusted significance level *α* = 0.0125). If the ANOVA showed no interaction between time and group, the main effects were directly analyzed; if an interaction existed, simple effect analysis for time and group was performed. A *p*-value <0.05 was considered statistically significant.

## 3 Results

### 3.1 General characteristics

During the 12-month recruitment period (June 2024 to May 2025), a total of 68 eligible patients were enrolled and randomly allocated at a 1:1 ratio, with 34 patients in the intervention group and 34 in the control group. All 68 patients completed the study and were included in the analysis. There were no statistically significant differences in general characteristics such as gender, age, wound type, and wound area between the two groups (*p* > 0.05), indicating comparability. Detailed data are presented in Table 1.

**Table 1:** Comparison of general characteristics between the two groups.

Characteristics	Intervention group (*n* = 34)	Control group (*n* = 34)	Test statistic	*p*-value
Gender			0.541	0.462^a^
Male	18 (52.9)	21 (61.8)		
Female	16 (47.1)	13 (38.2)		
Age (years, $\bar{x}$ ± *s*)	58.18 ± 16.96	59.88 ± 10.72	−0.494	0.623^b^
Wound type			1.282	0.922^a^
Diabetic foot	8 (23.5)	7 (20.6)		
Poor postoperative incision healing	9 (26.5)	12 (35.3)		
Abscess infection	5 (14.7)	3 (8.8)		
Non-healing after trauma	5 (14.7)	4 (11.8)		
Others	7 (20.6)	8 (23.5)		
Wound area [cm^2^, *M* (P25, P75)]	4 (1~13.8)	7.8 (3.5~40.125)	−1.927	0.054^c^

$\bar{x}$ ± *s* = mean ± standard deviation; *M* (P25, P75) = median (25th percentile, 75th percentile); Values in parentheses are percentages (%).

a Chi-square test.

b Independent samples *t*-test.

c Mann–Whitney *U* test.

### 3.2 Primary outcome

Repeated-measures analysis of variance (ANOVA) for wound treatment efficacy (Bates-Jensen Wound Assessment scores) showed that the main effects of time (*F* = 77.304, *p* < 0.001), group (*F* = 11.492, *p* = 0.001), and the time × group interaction (*F* = 4.075, *p* = 0.010) were all statistically significant. In non-parametric sensitivity analyses, no significant between-group difference was observed at baseline (Mann–Whitney *U* = 432.000, *p* = 0.072), while the intervention group showed progressively lower Bates-Jensen scores than the control group over follow-up; the differences at Weeks 1 and 2 were nominally significant (*p* = 0.026 and *p* = 0.015) but did not survive Bonferroni correction, and the Week-4 difference was highly significant (*U* = 293.500, *p* < 0.001). With the passage of time, the scores of both groups exhibited a decreasing trend, and the downward trend in the intervention group was more pronounced, suggesting that the medical consortium-based chain management protocol had a more significant promoting effect on wound recovery. Detailed data are shown in Table 2.

**Table 2:** Bates-Jensen wound assessment scores in the two groups at different time points [points, *M* (P25, P75)].

Group	Initial visit	Week 1	Week 2	Week 4	Time factor F/P	Group factor F/P	Time × Group F/P
Intervention group (*n* = 34)	30.00 (25.75, 31.00)	24.50 (22.00, 29.00)	19.00 (14.00, 25.00)	13.50 (6.00, 20.00)	77.304/<0.001	11.492/0.001	4.075/0.010
Control group (*n* = 34)	31.00 (27.00, 33.00)	27.50 (24.75, 31.00)	23.00 (20.75, 28.00)	21.00 (17.00, 24.00)			
Mann–Whitney *U*	432.000	397.000	380.000	293.500			
*Z* value	−1.797	−2.227	−2.432	−3.521			
*p*-value	0.072	0.026	0.015	<0.001			

Data are presented as median (P25, P75), as the Week-4 Bates-Jensen scores departed from normality (Shapiro–Wilk *p* < 0.05 in both groups). The two-way repeated-measures ANOVA was retained as the primary analysis given its robustness to moderate departures from normality with equal group sizes (*n* = 34 per group); the main effects of time, group, and the time × group interaction are shown in the last three columns. Mann–Whitney *U* tests at each time point were performed as non-parametric sensitivity analyses (Bonferroni-adjusted significance level *α* = 0.0125). *M* (P25, P75) = median (25th percentile, 75th percentile).

The median wound healing time was 30.5 (24.5~46.3) days in the intervention group and 52.5 (43.0~70.5) days in the control group, with a statistically significant difference between the two groups (*Z* = −3.343, *p* = 0.001), as presented in Table 3.

**Table 3:** Comparison of healing time in two groups [days, *M* (P25, P75)].

Group	Healing time	*Z*	*p*
Intervention group (*n* = 34)	30.5 (24.5~46.3)	−3.343	0.001
Control group (*n* = 34)	52.5 (43~70.5)		

### 3.3 Secondary outcomes

Regarding referral and consultation status, the implementation of the medical consortium-based chain management protocol is summarized in Table 4. During the study period, 7 patients (20.6%) were referred within the medical consortium, 10 in-hospital multidisciplinary team (MDT) consultations for chronic refractory wounds were initiated and completed, 6 inpatient consultations were performed, 12 home visits (home-based follow-up and wound care) were conducted, and 18 remote consultations were provided by wound and ostomy specialist nurses. All referral and consultation processes were conducted in accordance with the standardized chain management process, with no diagnostic and treatment delays. The individualized plans formulated by the MDT for cases such as diabetic foot and complex postoperative refractory wounds effectively addressed the limitations of single-department diagnosis and treatment.

**Table 4:** Implementation of the referral and consultation pathway in the intervention group (*n* = 34).

Indicator	Value
Patients referred within the medical consortium, *n* (%)	7 (20.6)
In-hospital MDT consultations for chronic refractory wounds, *n*	10
Inpatient consultations completed, *n*	6
Home visits (home-based follow-up and wound care), *n*	12
Remote (tele-) consultations, *n*	18

These indicators describe the implementation of the chain management protocol and were collected only in the intervention group, because the two-way referral and multidisciplinary consultation pathway constituted an integral component of the intervention. The control group received conventional care without consortium-based linkage, so corresponding indicators are structurally not applicable. Referral is reported as the number of patients; consultations and home visits are reported as the number of service events. These indicators are not mutually exclusive; a patient may receive multiple types of services, and a service may occur more than once for the same patient.

For patient satisfaction, the satisfaction rate was 97.1% (33/34) in the intervention group and 76.5% (26/34) in the control group, with a statistically significant difference between the two groups (Fisher’s exact test, *p* = 0.027). Patients in the intervention group reported that the seamlessly connected diagnosis and treatment services, professional wound care guidance, and convenient referral process brought by the chain management were the main reasons for the improved satisfaction. Detailed data are shown in Table 5.

**Table 5:** Comparison of patient satisfaction between the two groups [*n* (percentage, %)].

Groups	Satisfied	Dissatisfied	*χ*^2^ value (Pearson)	*p*-value (Fisher’s exact)
Intervention group (*n* = 34)	33 (97.1)	1 (2.9)	6.275	0.027
Control group (*n* = 34)	26 (76.5)	8 (23.5)		

*p*-values were calculated using Fisher’s exact test because the expected frequency in one cell was less than 5; the *χ*^2^ value (Pearson Chi-square test) is reported for reference.

## 4 Discussion

### 4.1 Core mechanisms of the medical consortium-based chain management protocol in promoting chronic wound healing

In this study, the median healing time in the intervention group was 30.5 days, which was significantly shorter than the 52.5 days in the control group (*p* = 0.001), and the Bates-Jensen scores in the intervention group were significantly lower over the follow-up period (time × group interaction *F* = 4.075, *p* = 0.010; Week-4 between-group difference *p* < 0.001). These findings are consistent with previous reports. A wound clinic-led chain management pathway has been reported to significantly improve wound healing status scores, reduce anxiety and depression, and enhance quality of life compared with conventional outpatient management (*p* < 0.05), consistent with the direction of benefits observed here (23). In addition, a multidisciplinary diagnosis and treatment model was shown to reduce the mean number of surgeries required for wound healing (2.1 ± 1.1 vs. 2.8 ± 1.6, *p* < 0.05) and improve patient satisfaction scores (96.5 vs. 91.1, *p* = 0.028) compared with non-MDT management (24), paralleling the higher satisfaction rate in our intervention group (97.1% vs. 76.5%, Fisher’s exact test, *p* = 0.027). Similarly, studies of multidisciplinary foot-care teams have reported reduced treatment costs and improved outcomes in diabetic foot disease (8) and lower in-hospital complications and mortality (18), and multidisciplinary approaches have also been effectively applied to hospital-acquired pressure injuries (19). Although differences in wound types, settings, and outcome definitions preclude direct numerical comparison of healing times across studies, the convergence of our results with these reports—across distinct outcome domains including healing status, surgical burden, satisfaction, and safety—supports the generalizability of the chain management approach. The protocol achieved this effect through dual mechanisms: First, the establishment of an in-hospital MDT (covering the Wound and Ostomy Clinic, Endocrinology Department, Orthopedics Department, etc.) and an out-of-hospital medical consortium collaborative network effectively integrated diagnosis and treatment resources, breaking the limitations of traditional “single-department practice.” Relevant studies on diabetic foot patient management have pointed out that MDT can comprehensively assess the patient’s systemic and local wound conditions, formulate individualized plans, and improve healing outcomes (8). In this study, for patients with comorbid diabetes and hypertension, collaborative interventions by the Endocrinology Department and Nutrition Department optimized key influencing factors such as blood glucose and nutrition, laying a foundation for wound repair. Second, the standardized chain process ensured the continuity of health services, with seamless connection from in-hospital initial assessment, MDT consultation, specialized intervention to out-of-hospital referral and home follow-up. This avoided service interruption and inconsistent management during cross-institutional and cross-departmental transfers under the traditional model (23), which is consistent with the proposed nursing concept of a “unified liaison point” in relevant wound care research (5). By clarifying responsible subjects and transfer standards, delays in service delivery were reduced, and the healing process was accelerated.

The full-chain management model in this study is analogous to the “real-time cross-institutional collaboration” concept of the Dr. LINK platform proposed in relevant research (25). The platform realizes multi-institutional information sharing and real-time consultation through a cloud-native architecture, while this protocol combines offline team establishment with online remote guidance, similarly breaking geographical and institutional barriers. Relevant studies have verified that a structured collaboration mechanism is the key to improving the accessibility of specialized diagnosis and treatment in environments with uneven resource distribution (25, 26). Compared with technologies focusing on local wound management proposed in existing research (27), this protocol emphasizes systematic health service optimization, integrating multi-dimensional resources including medical teams, care pathways, and training systems. It fundamentally solves the “fragmentation” problem in chronic wound management, confirming the clinical logic that “service process optimization is equally important as technical intervention”.

### 4.2 Value of the specialist nurse-led collaboration model in optimizing medical resources

Wound and ostomy specialist nurses assumed core coordinating responsibilities in the medical consortium-based chain management, including initial assessment, care planning, cross-institutional referral coordination, training of grassroots nurses, and remote guidance. The intervention group showed significant advantages in referral and consultation efficiency, fully verifying the value of this model. This is consistent with the conclusion of relevant research that advanced practice nurses can improve the medical experience by optimizing processes (26).

From the perspective of resource allocation, this model achieved efficient utilization through “technology diffusion to grassroots levels” and “two-way referral”: higher-level hospitals focused on MDT consultation and specialized intervention for complex wounds, while grassroots institutions undertook routine dressing changes and continuing care. This not only alleviated the service pressure on tertiary hospitals but also improved the service capacity of grassroots institutions (28). By conducting homogeneous training for nurses from 15 medical consortium units, this study effectively addressed the problem of grassroots institutions being “unable to receive or effectively treat patients”, which is similar to the application effect of the chronic wound hierarchical management information platform reported in relevant research (29). Meanwhile, remote guidance led by specialist nurses reduced the frequency of patient visits, lowered medical costs, and improved service accessibility (30). This is consistent with the core research logic that integrating multi-specialty resources can improve the healing rate of pressure ulcers in stroke patients and reduce patient burden (31), proving that specialist-led MDT collaboration is an effective path for optimizing resource allocation.

### 4.3 Unique advantages and practical significance of the medical consortium model in chronic wound management

The long-term nature and complexity of chronic wounds require cross-institutional and full-cycle service support, and the medical consortium structure provides an organizational foundation for this model as indicated in relevant research (32). This study vertically integrated resources from tertiary hospitals and grassroots institutions. Compared with the single in-hospital MDT model, it realized the standardization of out-of-hospital continuing care. For example, discharged patients were connected through specialist nurses in the medical consortium, ensuring the consistency of dressing change plans and the continuity of wound assessment. This avoided healing delays caused by improper self-care or inappropriate referral under the traditional model, echoing the “full-cycle closed-loop” concept of cloud-based management of chronic wounds in medical consortia proposed in existing research (33).

From a public health perspective, the promotion of this protocol is of great significance. With the aging population and the rising prevalence of chronic diseases, chronic wounds have become a severe public health challenge with high treatment costs as pointed out in relevant studies (25, 34). By improving healing efficiency and reducing complications and readmission rates, this protocol is expected to lower overall medical expenditures, which is consistent with the research finding that MDT-based diabetic foot care can reduce treatment costs (8). Meanwhile, standardized training enhances the “self-sustaining service capabilities” of grassroots medical institutions, providing a practical path for the implementation of hierarchical diagnosis and treatment. This aligns with the research goal of empowering resource-poor areas through technical means in relevant medical platform studies (25), offering support for addressing uneven resource distribution and improving medical service equity.

### 4.4 Study limitations and future prospects

This study has certain limitations: First, the study subjects were recruited from a tertiary hospital in Nanchang and its affiliated medical consortium units, resulting in limited sample representativeness and potential selection bias. Future multi-center, large-sample health services research is needed to verify the universality and adaptability of the protocol in regions with different economic levels and resource allocations. Second, due to the nature of the service intervention, blinding was not feasible, and no separate technical intervention control group was set up, making it impossible to quantify the independent effects of process optimization and technical intervention. Subsequent factorial design studies can clarify the role of each intervention component. Third, the post-discharge follow-up period was relatively short, failing to fully reveal long-term benefits. Follow-up should be extended to 6 months to 1 year to evaluate its role in preventing recurrence and reducing long-term complications. Additionally, systematic analysis of health economic benefits was not conducted; future cost-effectiveness analysis can quantify its economic value, providing a basis for large-scale promotion. In addition, referral and consultation data were collected only for the intervention group, as the two-way referral and multidisciplinary consultation pathway was an integral component of the intervention; specialist visits self-initiated by control-group patients were not systematically recorded, precluding a between-group comparison of these implementation indicators. Moreover, patient satisfaction was measured with a self-designed, study-specific questionnaire that has not undergone formal psychometric validation; future studies should employ or develop validated satisfaction instruments.

Future research can be improved in four aspects: First, integrate digital technologies to build an information management system incorporating wound image upload, AI-assisted assessment and remote consultation, so as to improve the collaboration efficiency and data security within the medical consortium. This approach has been verified in relevant research on digitally enabled home-based wound care, where its AI assessment and 3D reconstruction technologies can significantly enhance the accuracy of wound assessment and the efficiency of remote collaboration (35). Second, expand the coverage of the medical consortium to include community health service centers and home care institutions, improving the “hospital-community-home” three-level network proposed in relevant research (5, 36). Third, develop personalized pathways for different types of chronic wounds and combine local wound management technologies reported in existing research to enhance targeting (27). Fourth, strengthen the cultivation of patients’ self-management capabilities and improve compliance through health education to promote long-term rehabilitation.

### 4.5 Implications and recommendations for clinical practice

This study confirms that the medical consortium-based chain management protocol is scientifically effective, providing important implications for health service delivery and management. At the management level, hospital administrators should promote the establishment of a specialist nurse-led cross-institutional collaboration mechanism, integrate informatization and standardization into daily workflows (37), and strengthen the integration of medical consortium resources and information sharing, unifying evaluation standards and care protocols (28). This is consistent with the approach of standardized data interoperability applied in relevant medical platform research (25). At the practical level, specialist nurses should play a core coordinating role, strengthen the linkage between in-hospital MDT and grassroots institutions, ensure the continuity and homogeneity of care (37), and attach importance to the training and empowerment of grassroots nurses to provide technical support for hierarchical diagnosis and treatment. Healthcare providers should emphasize MDT collaboration, jointly formulate comprehensive plans with specialties such as endocrinology and nutrition, and combine advanced technologies to improve healing effects. Patients should actively participate in self-management, follow guidance for self-care and regular follow-up, and improve compliance.

## 5 Conclusion

This health services intervention study demonstrates that the medical consortium-based chain management protocol effectively integrates multidisciplinary resources to shorten healing times for chronic refractory wounds, while significantly boosting patient satisfaction and inter-institutional collaboration efficiency. Led by specialist nurses, this model transcends geographical and institutional barriers through standardized care processes, optimizes health resource allocation, and enhances the equity and homogenization of wound care services. It delivers a feasible, scalable approach to the systematic management of chronic wounds within the health service system, furnishes practical support for the implementation of hierarchical diagnosis and treatment, and holds important implications for the management of other chronic conditions and public health practice.

## Data Availability

The raw data supporting the conclusions of this article will be made available by the authors, without undue reservation.

## References

[1] Martinengo L Olsson M Bajpai R Soljak M Upton Z Schmidtchen A . Prevalence of chronic wounds in the general population: systematic review and meta-analysis of observational studies. Ann Epidemiol. (2019) 29:8–15. doi: 10.1016/J.ANNEPIDEM.2018.10.005 30497932

[2] Han G Ceilley R. Chronic wound healing: a review of current management and treatments. Adv Ther. (2017) 34:599–610. doi: 10.1007/S12325-017-0478-Y PMC5350204 28108895

[3] Zhao R Liang H Clarke E Jackson C Xue M. Inflammation in chronic wounds. Int J Mol Sci. (2016) 17:2085. doi: 10.3390/IJMS17122085 PMC5187885 27973441

[4] Sengul T Kirkland-Kyhn H Karadag A. Chronic wounds and dressings: an overview of management and effectiveness. Nurs Clin North Am. (2025) 60:1–13. doi: 10.1016/j.cnur.2024.08.008 39884782

[5] Fitzpatrick S Hawkins S Dunlap E Nagarsheth K. Nurse driven outpatient wound center: reducing readmission with wound care excellence. J Vasc Nurs. (2022) 40:100–4. doi: 10.1016/j.jvn.2022.05.002 35750372

[6] Olsson M Järbrink K Divakar U Bajpai R Upton Z Schmidtchen A . The humanistic and economic burden of chronic wounds: a systematic review. Wound Repair Regen. (2019) 27:114–25. doi: 10.1111/wrr.12683 30362646

[7] Hicks CW Canner JK Mathioudakis N Lippincott C Sherman RL Abularrage CJ. Incidence and risk factors associated with ulcer recurrence among patients with diabetic foot ulcers treated in a multidisciplinary setting. J Surg Res. (2020) 246:243–50. doi: 10.1016/j.jss.2019.09.025 31610352

[8] Joret MO Osman K Dean A Cao C van der Werf B Bhamidipaty V. Multidisciplinary clinics reduce treatment costs and improve patient outcomes in diabetic foot disease. J Vasc Surg. (2019) 70:806–814.e1. doi: 10.1016/j.jvs.2018.11.032 30850290

[9] National Bureau of Statistics of China. Statistical Communiqué on the 2023 National Economic and Social Development [Internet]. Beijing: NBS (2024).

[10] Sun H Saeedi P Karuranga S Pinkepank M Ogurtsova K Duncan BB . IDF diabetes atlas: global, regional and country-level diabetes prevalence estimates for 2021 and projections for 2045. Diabetes Res Clin Pract. (2022) 183:109119. doi: 10.1016/j.diabres.2021.109119 PMC11057359 34879977

[11] Fu X Sheng Z Cherry GW Li Q. Epidemiological study of chronic dermal ulcers in China. Wound Repair Regen. (1998) 6:21–7. doi: 10.1046/j.1524-475x.1998.60105.x 9776847

[12] Jiang Y Huang S Fu X Liu H Ran X Lu S . Epidemiology of chronic cutaneous wounds in China. Wound Repair Regen. (2011) 19:181–8. doi: 10.1111/j.1524-475X.2010.00666.x 21362085

[13] Cheng B Jiang Y Fu X Hao D Liu H Liu Y . Epidemiological characteristics and clinical analyses of chronic cutaneous wounds of inpatients in China: prevention and control. Wound Repair Regen. (2020) 28:623–30. doi: 10.1111/wrr.12825 32585756

[14] Li H. Editorial: wound repair: establishment and development of a new discipline in China. Front Surg. (2023) 10:1207709. doi: 10.3389/fsurg.2023.1207709 PMC10227557 37260596

[15] Jiang Q Dumville JC Cullum N Pan J Liu Z. Epidemiology and disease burden of complex wounds for inpatients in China: an observational study from Sichuan province. BMJ Open. (2020) 10:e039894. doi: 10.1136/bmjopen-2020-039894 PMC7651747 33158828

[16] Wang FF Qu H Liu XY. Practice of chain management in continuous nursing under the medical consortium cooperation model. J Nurs Sci. (2021) 36:1–4. doi: 10.3870/j.issn.1001-4152.2021.19.001

[17] Lu SX Sun J. Suggestions on further promoting the construction of “medical consortium” and continuously improving the level of community medical and health services. Chin Community Doctors. (2016) 32:189–90.

[18] Meloni M Andreadi A Bellizzi E Giurato L Ruotolo V Romano M . A multidisciplinary team reduces in-hospital clinical complications and mortality in patients with diabetic foot ulcers. Diabetes Metab Res Rev. (2023) 39:e3690. doi: 10.1002/dmrr.3690 37422897

[19] Roderman N Wilcox S Beal A. Effectively addressing hospital-acquired pressure injuries with a multidisciplinary approach. HCA Healthcare J Med. (2024) 5:577–86. doi: 10.1016/j.hcaj.2024.02.004 PMC11547285 39524937

[20] Zang D Jiang SQ Meng Y Guo CL Zhong Y. Application and effect evaluation of full-cycle breastfeeding management for high-risk infants based on chain theory. Chin Nurs Manag. (2025) 25:80–5. doi: 10.3969/j.issn.1672-1756.2025.01.016

[21] Gu J Zhang YY. Effect of multidisciplinary joint health education based on chain management model on medication compliance of outpatients with hypertension. Nurs J Chin People’s Liber Army. (2019) 36:54–7. doi: 10.3969/J.ISSN.1008-9993.2019.05.015

[22] Macfarlane SM Zhao SX Lafrenz JO Nagaratnam MV Tchen A Linton CE . Effect of a multidisciplinary team approach on the management of diabetic foot ulcers on the central coast: a review of the Gosford hospital high-risk foot clinic. Int Wound J. (2024) 21:e14570. doi: 10.1111/iwj.14570 PMC10822746 38379247

[23] Zheng XL Hu XL. Application analysis of wound clinic-led chain management pathway in chronic wounds. Acta Acad Med Wannan. (2024) 43:402–5. doi: 10.3969/j.issn.1002-0217.2024.04.013

[24] Wang LW Liu BC Qu YY Wu CY Tian Y. Clinical application of multidisciplinary diagnosis and treatment model in the diagnosis and treatment of chronic refractory wounds. J Peking Univ Health Sci. (2025) 57:185–92. 10.19723/j.issn.1671-167X.2025.01.028 PMC11759794 39856526

[25] Lee HY Chae SH Kim HJ Lee J Moon H Lee Y . Implementation and assessment of ‘Dr. LINK’ platform: a remote collaborative care platform for trauma and hyperbaric oxygen therapy in underserved areas. Appl Sci. (2025) 15:11637. doi: 10.3390/app152411637

[26] Smigorowsky MJ Norris CM McMurtry MS Tsuyuki RT. Measuring the effect of nurse practitioner (NP)-led care on health-related quality of life in adult patients with atrial fibrillation: study protocol for a randomized controlled trial. Trials. (2017) 18:364. doi: 10.1186/s13063-017-2111-4 PMC5543536 28774317

[27] Milcheski DA Clivatti GM Santos Junior RA González CV Monteiro Jr AA Gemperli R. Effectiveness of negative-pressure wound therapy with instillation compared to standard negative-pressure wound therapy and traditional gauze layer dressing for the treatment of acute traumatic wounds. J Plast Reconstr Aesthet Surg. (2025) 100:208–18. doi: 10.1016/j.bjps.2024.11.005 39644780

[28] Li Z Fang H. Practical experience and suggestions on the hierarchical diagnosis and treatment system in Chongqing. Health Econ Res. (2025) 42:37–41. doi: 10.3969/J.ISSN.1004-7778.2025.03.008

[29] Xu J Kong XR Jiang QX Liu X Zhang DJ Tang LL . Construction and application of hierarchical management information platform for chronic wounds. Chin Nurs Manag. (2022) 22:760–3. doi: 10.3969/J.ISSN.1672-1756.2022.05.024

[30] Kilfoy A Chu C Krisnagopal A McAtee E Baek S Zworth M . Nurse-led remote digital support for adults with chronic conditions: a systematic synthesis without meta-analysis. J Clin Nurs. (2025) 34:715–36. doi: 10.1111/jocn.17226 PMC11808464 38894583

[31] Gu YH Wang X Sun SS. Benefits of multidisciplinary collaborative care team-based nursing services in treating pressure injury wounds in cerebral infarction patients. World J Clin Cases. (2022) 10:43–50. doi: 10.12998/wjcc.v10.i1.43 PMC8727259 35071504

[32] Sun HL Lan MJ Wang H Chen HT Yu HQ. Qualitative study on the barriers to linked management of chronic wound care within medical consortia. Chin J Nurs. (2025) 60:2246–51.

[33] Zhang ZP Zhang W Xie P Tang YJ. Application research of cloud-based management model for chronic wounds in medical consortia based on internet hospitals. Mod Clin Nurs. (2024) 23:82–7. doi: 10.3969/j.issn.1671-8283.2024.03.012

[34] Lindholm C Searle R Nelson E. Wound management for the 21st century: combining effectiveness and efficiency. Int Wound J. (2016) 13:5–15. doi: 10.1111/iwj.12551 PMC7949725 27460943

[35] Chen L Zhuo X Cai H Zeng P Chen H Chen G. A digitally enabled home-based wound care program using the PEDALs model: a mixed-methods study protocol. Front Public Health. (2025) 13:1591187. doi: 10.3389/fpubh.2025.1591187 PMC12178876 40547473

[36] Seaton PCJ Cant RP Trip HT. Quality indicators for a community-based wound care centre: an integrative review. Int Wound J. (2020) 17:587–600. doi: 10.1111/iwj.13271 PMC7949261 32030879

[37] Li HY Sun JN Zhang Q . Application of wound specialist nurse-led chain management pathway in cancerous wounds of breast cancer patients. Chin Nurs Manag. (2022) 22:1121–6. doi: 10.3969/j.issn.1672-1756.2022.08.011

